# Identification of a dominant gene in *Medicago truncatula* that restricts nodulation by *Sinorhizobium meliloti* strain Rm41

**DOI:** 10.1186/1471-2229-14-167

**Published:** 2014-06-16

**Authors:** Jinge Liu, Shengming Yang, Qiaolin Zheng, Hongyan Zhu

**Affiliations:** 1 Department of Plant and Soil Sciences, University of Kentucky, Lexington, KY 40546, USA

**Keywords:** Legume, *Medicago truncatula*, Nodulation specificity, Nitrogen fixation

## Abstract

**Background:**

Leguminous plants are able to form a root nodule symbiosis with nitrogen-fixing soil bacteria called rhizobia. This symbiotic association shows a high level of specificity. Beyond the specificity for the legume family, individual legume species/genotypes can only interact with certain restricted group of bacterial species or strains. Specificity in this system is regulated by complex signal exchange between the two symbiotic partners and thus multiple genetic mechanisms could be involved in the recognition process. Knowledge of the molecular mechanisms controlling symbiotic specificity could enable genetic improvement of legume nitrogen fixation, and may also reveal the possible mechanisms that restrict root nodule symbiosis in non-legumes.

**Results:**

We screened a core collection of *Medicago truncatula* genotypes with several strains of *Sinorhizobium meliloti* and identified a naturally occurring dominant gene that restricts nodulation by *S. meliloti* Rm41. We named this gene as *Mt-NS1 (for M.truncatulan*odulation *s*pecificity 1). We have mapped the *Mt-NS1* locus within a small genomic region on *M. truncatula* chromosome 8. The data reported here will facilitate positional cloning of the *Mt-NS1* gene.

**Conclusions:**

Evolution of symbiosis specificity involves both rhizobial and host genes. From the bacterial side, specificity determinants include Nod factors, surface polysaccharides, and secreted proteins. However, we know relatively less from the host side. We recently demonstrated that a component of this specificity in soybeans is defined by plant NBS-LRR resistance (*R*) genes that recognize effector proteins delivered by the type III secretion system (T3SS) of the rhizobial symbionts. However, the lack of a T3SS in many sequenced *S. meliloti* strains raises the question of how the specificity is regulated in the *Medicago*-*Sinorhizobium* system beyond Nod-factor perception. Thus, cloning and characterization of *Mt-NS1* will add a new dimension to our knowledge about the genetic control of nodulation specificity in the legume-rhizobial symbiosis.

## Background

Legumes are able to enter into a symbiotic relationship with nitrogen-fixing soil bacteria, collectively called rhizobia. The symbiosis culminates in the formation of the root nodule, which provides an optimal environment for the bacteria to fix atmospheric nitrogen for use by the plant. It was estimated that the legume-rhizobial symbiosis can fix more than half of the amount of nitrogen produced by the chemical fertilizer industry [1].

The legume-rhizobial interaction begins with a molecular dialogue between the two symbiotic partners [2]. Flavonoid compounds released into the rhizosphere by the legume roots attract rhizobia and induce the expression of a set of bacterial genes, known as the *nod* genes [3-5]. The enzymes encoded by the *nod* genes enable the synthesis and secretion of bacterial lipo-chitooligosaccharides known as nodulation (Nod) factors [2,6]. Perception of Nod factors by the cognate host receptors in turn activates a suite of host responses that are essential for accommodation of bacterial invasion [7-10]. One of the earliest plant responses is the curling of the root hairs which traps rhizobial bacteria within a structure called the colonized curled root hair [11,12]. It is within these trap sites that infection threads are initiated and extended, through which the bacteria are transported and ultimately released to the dividing cortical cells named the nodule primordium [5]. Within these nodule cells, the bacteria are enclosed in host-membrane-bound compartments known as symbiosomes and differentiate into the nitrogen*-*fixing bacteroids [13,14]. During the last decade, a number of plant genes have been identified in *Medicago truncatula* and *Lotus japonicus* that are required for rhizobial infection and nodule development [15,16]. The cloning and characterization of these genes has revealed the nodulation signaling pathway that is conserved in legumes [17].

A significant property of the legume-rhizobial symbiosis is its high level of specificity [5,18-20]. Beyond the specificity for the legume family, individual legume species/genotypes can only interact with certain restricted group of bacterial species or strains. Specificity in this system can take place at multiple stages of the interaction, ranging from initial bacterial infection and nodulation (nodulation specificity) to late nodule development associated with nitrogen fixation efficiency (nitrogen fixation specificity) [19]. Understanding the molecular mechanisms underlying symbiotic specificity will enable genetic manipulation of the host or bacteria in order to enhance the agronomic potential of the root nodule symbiosis. This can be achieved either by extending the host range of bacterial strains with high nitrogen fixation efficiency or conversely, by excluding indigenous soil strains that are highly competitive for nodulation but with low nitrogen-fixing capability [20].

The establishment of a root nodule symbiosis involves the exchange of a series of signals between the plant and bacteria. Accordingly, genetic control of symbiosis specificity is complex and multiple mechanisms could be involved [19]. It is widely believed that the host range is mainly determined by specific recognition of bacterial Nod factors by the cognate host receptor(s) [5,21-26]. However, natural variation in Nod factor receptors that causes changed specificity has rarely been documented. In contrast, a number of dominant genes have been identified in soybeans and other legumes that restrict nodulation with specific rhizobial strains [20,27-30]. The dominant nature of these genes resembles ‘gene-for-gene’ resistance against plant pathogens [19,20,31]. We have demonstrated that a component of this specificity in soybeans is defined by plant NBS-LRR resistance genes that recognize effector proteins delivered by the type III secretion system (T3SS) of the rhizobial symbionts [20]. However, the lack of a T3SS in many sequenced *Sinorhizobium meliloti* strains raises the question of how the specificity is regulated in the *Medicago*-*Sinorhizobium* system beyond Nod-factor perception. To address this question, we screened a core collection of *M. truncatula* genotypes with several strains of *S. meliloti* and identified a naturally occurring dominant gene, *Mt-NS1*, in *M. truncatula* that restricts nodulation by *S. meliloti* Rm41. Genetic mapping experiments indicated that *Mt-NS1* is not likely a typical *R* gene. Thus, cloning and characterization of *Mt-NS1* will add a new dimension to our knowledge about the genetic control of nodulation specificity in the legume-rhizobial symbiosis.

## Results and discussion

### Natural variation in symbiosis specificity in *M. truncatula*

**Figure 1:**
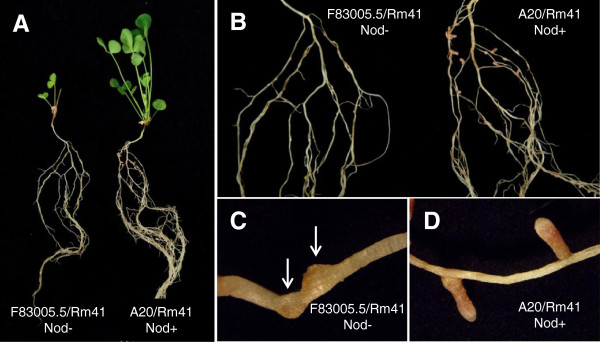
**Nodulation and growth phenotypes of *M. truncatula *plants after inoculation with *S. meliloti *strain Rm41. A**, F83005.5 and A20 lines after inoculation by *S. meliloti* strain Rm41. A20 (right) grew normally under nitrogen-free conditions, whereas F83005.5 (left) can hardly survive under the same conditions. **B**, Nodulation phenotypes of the same plants in panel A. A20 roots formed nitrogen-fixing root nodules (right), while F83005.5 cannot nodulate with Rm41. **C** and **D**, a closer look at the nodulation phenotypes of A20 and F83005.5, showing fully developed functional nodules on the roots of A20 **(D)** and the nodule primordial (arrows) formed on the roots of F83005.5 **(C)**.

It had previously been reported that the *M. truncatula* plants showed differential nitrogen fixation efficiency when inoculated with different rhizobial strains [32-35]. However, to our knowledge, natural variation in nodulation specificity (i.e.*,* Nod + vs. Nod- phenotypes) has not been well-documented. To gain a better understanding of the genetic mechanisms underlying symbiosis specificity in the *M. truncatula*-*Sinorhizobium* interaction, we screened a core collection of 31 *M. truncatula* genotypes using the *S. meliloti* strains NGR34, NGR247, and Rm41. These plant genotypes capture a wide range of genetic diversity present in natural populations of *M. truncatula*[36]. This experiment revealed tremendous variation in nodulation capacity and nitrogen fixation specificity between different genotype-rhizobial combinations (Table 1). In particular, this screen revealed that Rm41 was unable to nodulate the plant genotypes F83005.5 and Turkey (Figure 1), while the same plant genotypes nodulated normally with other *S. meliloti* strains. Thus, we postulate that there exist host genes that control strain-specific nodulation in *M. truncatula*. For genetic analysis of the nodulation specificity in this system, we chose to focus on F83005.5 because Turkey was not compatible when crossed with several other *M. truncatula* genotypes.

**Table 1:** Natural variation in symbiosis specificity in M. truncatula*

**Plant genotypes**	**Rhizobial strains**			**Plant genotypes**	**Rhizobial strains**		
	**NGR247**	**NGR34**	**Rm41**		**NGR247**	**NGR34**	**Rm41**
A17	Fix-	Fix+	Fix-	ESP165-D	Fix-	Fix+	Fix-
A20	Fix+	Fix-	Fix+	F11.005-E	Fix+	Fix-	Fix+
Borung	Fix-	Fix-	Fix+	F11.013-3	Fix+	Fix-	Fix+
Caliph-A	Fix+	Fix-	Fix+	F20047-A	Fix+	Fix-	Fix+
Cyprus-C	Fix+	Fix+	Fix+	F20061-A	Fix+	Fix-	Fix+
DZA055-H	Fix-	Fix-	Fix+	F20089-B	Fix+	Fix-	Fix+
DZA105-1	Fix+	Fix+	Fix+	F34.042-D	Fix-	Fix-	Fix+
DZA220	Fix+	Fix+	Fix+	F83005.5	Fix-	Fix-	**Nod-**
DZA222	Fix-	Fix+	Fix+	GRC020B	Fix+	Fix+	Fix+
DZA233-4	Fix+	Fix-	Fix+	GRC043-1	Fix-	Fix+	Fix+
DZA315-16	Fix-	Fix+	Fix+	GRC064-B	Fix+	Fix-	Fix+
DZA327-7	Fix+	Fix+	Fix+	Harbinger	Fix+	Fix+	Fix+
DZA045.5	Fix-	Fix-	Fix+	Paraggio	Fix+	Fix-	Fix+
ESP105-L	Fix-	Fix+	Fix-	Sephi-A	Fix-	Fix-	Fix+
ESP158-A	Fix-	Fix+	Fix-	Turkey	Fix-	Fix-	**Nod-**
ESP159-11	Fix-	Fix+	Fix-				

*Fix + = pink cylindrical nodules colonized by bacteria able to fix nitrogen; Fix- = small, white and round nodules colonized by bacteria unable to fix nitrogen; Nod- = roots unable to form nodules.

### Rm41 induced root hair curling and nodule primordium formation but failed to infect the roots of F83005.5

**Figure 2:**
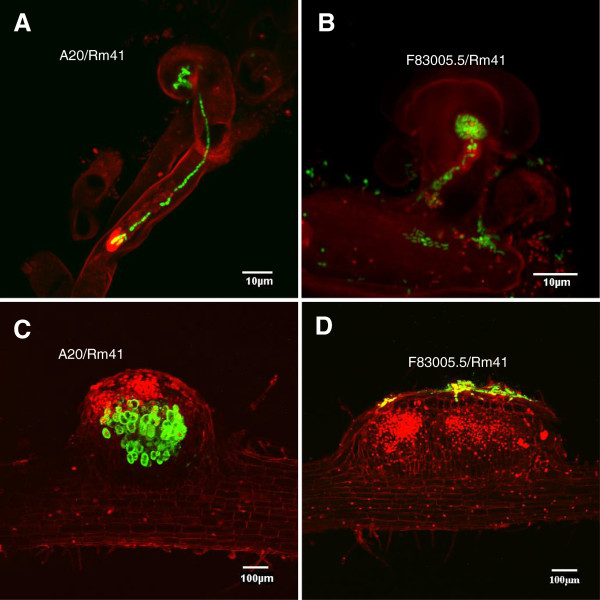
**Fluorescence microscopy analyses of infection process of compatible and incompatible interactions between *M. truncatula *plants and Rm41.** All images are composite images of GFP-expressing Rm41 cells (green) and root cells (red). **A**, Typical infection thread formed by compatible interaction between A20 and Rm41. The infection thread extends from the colonized, curled root hair to the base of the root hair cell. **B**, In the incompatible interaction between F83005.5 and Rm41, the bacteria can normally colonize the curled root hairs but typical infection threads cannot be detected. Occasionally, we can detect aborted, aberrant infection threads present on the F83005.5 roots. **C**, The nodule primordium on the A20 roots contained bacteria, while the nodule primordia on the F83005.5 roots **(D)** contained no bacteria despite frequent presence of bacterial colonies on the epidermal surface of the nodule primordia.

*S. meliloti* Rm41 is a wild-type strain originally isolated from alfalfa nodules in Hungary [37]. Specifically, this strain contains a strain-specific K antigen (also known as capsular polysaccharides or KPS) which is able to compensate for the lack of exopolysaccharides (EPS) production that is generally required for successful invasion of indeterminate nodules on the alfalfa roots [12,38,39]. The Rm41 genome has recently been sequenced, consisting of a 3.68-Mb chromosome, two symbiotic plasmids (1.56-Mb pSymA and 1.66-Mb pSymB), and a 246-kb nonsymbiotic plasmid pRme41a [40]. It is noteworthy that, similar to many other sequenced *S. meliloti* strains, the genome of Rm41 does not possess genes encoding a type III secretion system (T3SS) that delivers effector proteins into the host cell [40].To examine the infection process, we used an Rm41 strain that constitutively expresses the green fluorescent protein (GFP) from a stably maintained plasmid vector pHC60. While inoculation of F83005.5 by Rm41 failed to induce root nodule formation, the rhizobial strain was able to induce both root hair curling and nodule primordium formation (Figure 2), suggesting that the early responses of Nod-factor perception were not affected. The bacteria can normally colonize the curled root hairs and occasionally, we can detect aborted, aberrant infection threads present on the F83005.5 roots (Figure 2B). However, in contrast to the compatible interaction between Rm41 and A20 (Figure 2A), normal infection threads were never observed on the roots of F83005.5. In consistent with these observations, the nodule primordia on the F83005.5 roots contained no bacteria despite frequent presence of bacterial colonies on the epidermal surface of the nodule primordia (Figure 2D). Due to a lack of infection, cortical cell division on the F83005.5 roots ceased at an early stage, whereas on A20 roots, infected nodule primordia were readily formed within 4–5 days post inoculation (Figure 2C). Based on these observations, we conclude that the restriction of nodulation by Rm41 in F83005.5 was due to block of bacterial infection rather than a failure in Nod factor perception.

### Exopolysaccharides production of Rm41 is required for establishing efficient symbiotic interactions with *M. truncatula*

Rhizobial surface polysaccharides, such as exopolysaccharides (EPS), capsular polysaccharides (KPS), and lipopolysaccharides (LPS), form complex macromolecular structures at the bacterium–plant interface and play important roles in establishment of the symbiotic relationship between the host and bacteria [12,19,38,41,42]. In particular, these polysaccharides have been implicated in playing a key role in facilitating infection thread initiation and extension in the alfalfa-*S. meliloti* interactions [38,43,44]. Despite being symbiotically important, these molecules, as common microbe-associated molecular patterns (MAMPs) of the rhizobial bacteria, may also trigger defense responses upon recognition by the cognate host pattern recognition receptors (PRRs) and thus are possibly associated with symbiosis specificity.

We inoculated the A20 (Nod+), DZA045.5 (Nod+), and F83005.5 (Nod-) plants with Rm41 mutants that are defective in production of various components of surface polysaccharides. The mutants used in this study included AK631, an *exoB* mutant of Rm41 deficient in EPS production (EPS-); PP4709, an *rkp1* mutant of RM41 deficient in production of KPS (KPS-), and PP674, an *rkp1* mutant of AK631 deficient in production of both EPS and KPS (EPS-KPS-) (Table 2). Our data showed that PP4709 (KPS-) behaved similar to the wild-type strain Rm41 and can normally nodulate A20 and DZA045.5, suggesting that KPS production of Rm41 is not required for nodulation with the *M. truncatula* genotypes A20 and DZA045.5. In contrast, AK631 (ESP-) and PP674 (EPS-KPS-) were not able to nodulate DZA045.5, and AK631 only induced the formation of a few Fix- nodules on the A20 roots, which suggested that EPS production plays an important role in establishing an efficient symbiosis with *M. truncatula* and their role can’t be complemented by KPS. Our data are consistent with that reported by Simesk et al. [35].

**Table 2:** **Nodulation phenotypes of *M. truncatula *genotypes with Rm41 mutants**

	**F83005.5**	**DZA045.5**	**A20**
Rm41	Nod-	Fix+	Fix+
AK631 (EPS-)	Nod-	Nod-	Fix-*
PP4709 (KPS-)	Nod-	Fix+	Fix+
PP674 (EPS-KPS-)	Nod-	Nod-	Nod-

*Only a few non functional nodules were formed.

Neither single nor double mutants of Rm41 could nodulate F83005.5, which appears to suggest that the incompatibility between F83005.5 and Rm41 is not associated with EPS and KPS production. However, we can’t exclude the possibility that EPS is required for nodulation in the compatible interaction but also essential for eliciting defense responses in the F83005.5 background. This scenario is similar to the T3SS of pathogenic bacteria, for which T3SS is required for causing disease in susceptible hosts and for eliciting the hypersensitive response in resistant hosts, and defects in the T3SS renders a bacterium non-pathogenic [45].

### The restriction of nodulation by Rm41 in F83005.5 is controlled by a single dominant gene

**Figure 3:**
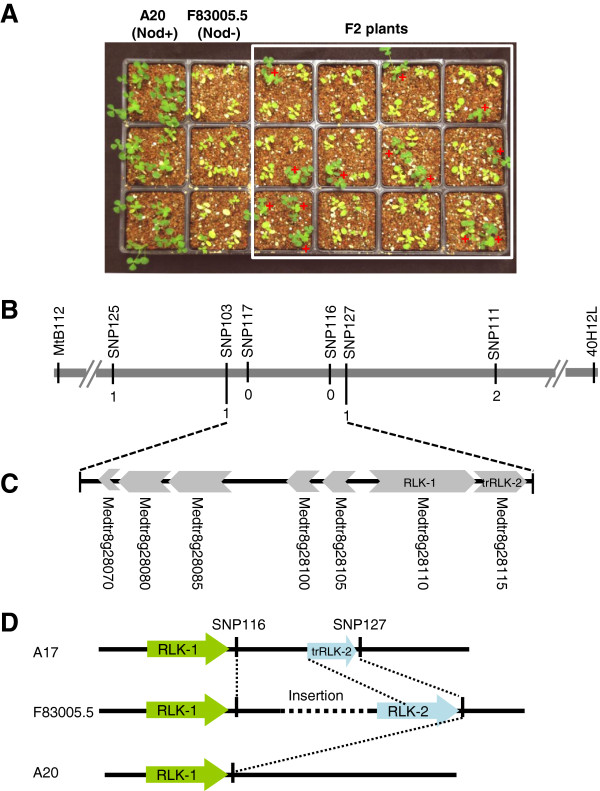
**Genetic mapping of *Mt-NS1*. A**, Growth phenotypes of A20 (Nod+, *ns1/ns1*), F83005.5 (Nod-, *NS1/NS1*), and a subpopulation of F2 plants three weeks post inoculation with *S. meliloti* Rm41. Nodulated plants are green and healthy, whereas the non-nodulated plants showed nitrogen starvation symptoms with yellowish leaves under the nitrogen-free condition. **B**, Fine mapping of *Mt-NS1*. The *Mt-NS1* locus was delimited to a genomic region between markers SNP103 and SNP127. Numbers indicate the number of recombination breakpoints separating the marker from *Mt-NS1* based on genotyping ~3,900 F2 plants. **C**, Annotation of the 50-kb genomic DNA of Jemalong A17 (Nod + Fix-, *ns1/ns1*) that covers the candidate gene region identifies 7 putative genes (Medtr8g28070-Metr8g28115). **D**, Identification of an insertion/deletion polymorphism between the two copies of the receptor-like kinases (Metr8g28110 and Metr8g28115) among the genomes of Jemalong A17, A20, and F83005.5.

For genetic analysis of the nodulation specificity, we used an F2 population derived from the cross between the two *M. truncatula* genotypes A20 and F83005.5. A20 showed Nod + Fix + phenotype when inoculated with Rm41. From a total of 2,623 inoculated F_2_ plants, 686 plants nodulated, which fits the 3:1 (non-nodulation to nodulation) ratio (χ^2^ = 1.86, *df* = 1, *P* = 0.17; Figure 3A), suggesting that the restriction of nodulation by Rm41 in F83005.5 is controlled by a single dominant gene. We named this gene as *Mt-NS1* (for *M. truncatula* nodulation specificity 1). Consistent with the cytological studies described above, the dominant nature of this gene indicates that the non-nodulation phenotype of F83005.5 is not due to a failure in Nod factor perception but resembles ‘gene-for-gene’ resistance against pathogen infections.

### Genetic mapping of *Mt-NS1*

Genetic mapping of *Mt-NS1* was initially carried out in an F2 population derived from the cross between A20 and F83005.5, which allowed us to map the *Mt-NS1* locus on chromosome 8, defined by the flanking markers MtB112 and 40H12L (Figure 3B). However, the extremely low levels of sequence polymorphisms between A20 and F83005.5 prevented us from using this population for fine mapping of the locus. To solve this problem, we developed another F2 population derived from the cross of DZA045.5 (Nod + Fix+) and F83005.5.

We took advantage of the availability of the *M. truncatula* genome sequence [46] to develop high-density SNP (single nucleotide polymorphism) markers for fine mapping of the *Mt-NS1* locus. SNPs were genotyped either by converting to CAPS (cleaved amplified polymorphic sequences) markers or by direct sequencing. Phenotyping and genotyping a total of 3,900 F2 plants using the SNP markers allowed us to delimit the *Mt-NS1* locus between SNP103 and SNP127 (Figure 3B), which span ~50 kb based on the genome sequence of Jemalong A17 (Nod + Fix-). The 50-kb genomic sequence of Jemalong A17 contains at least six predicted genes based on the *Medicago truncatula* genome release version 4.0 (http://www.jcvi.org/medicago) (Figure 3C). These genes include Medtr8g028070, a ferric-chelate reductase-like protein; Medtr8g028080, an RNase T2 family protein; Medtr8g028085 and Medtr8g028100, two highly conserved proteins with unknown function; Medtr8g028105, an A/G-specific adenine DNA glycosylase-like protein; and Medtr8g028110, a leucine-rich repeat receptor-like kinase (LRR-RLK). In particular, the LRR-RLK (Medtr8g028110; here we call it as RLK-1) is structurally similar to the DMI2/SYMRK orthologs in legumes that are required for both rhizobial and mycorrhizal symbioses, which consists of an N-terminal signal peptide, an extracellular malectin-like domain comprising three leucine-rich repeats, a transmembrane domain, and an intracellular protein kinase domain [47,48]. In addition, there exists a tandem duplication*–*truncation of the RLK-1 gene (annotated as Medtr8g028115; here we call it as trRLK-2) (Figure 3C). As described below, the presence of a large insertion/deletion polymorphism around the *Mt-NS1* locus between F83005.5 and Jemalong A17 complicated the prediction of candidate genes.

### Identification of an insertion/deletion polymorphism around the *Mt-NS1* locus

Based on our knowledge of the signaling pathways in pathogenic and symbiotic plant-microbe interactions, we had assumed that RLK-1 was a strong candidate gene for *Mt-NS1*. This gene may serve as a receptor that directly or indirectly perceives a yet unknown bacterial signal, which in turn triggers host defense responses and blocks bacterial infection. Support of this hypothesis also came from the fact that this gene showed root-specific expression (data not shown). However, we did not detect any expression- and sequence-level polymorphisms between the F83005.5 and A20 alleles of RLK-1. Since the phenotype of the reference genotype A17 is Nod + Fix- (*ns1/ns1*), it is possible that *ns1* represents a null allele in A17. Full cDNA amplification identified two copies of LRR-RLK genes in F83005.5; one corresponds to RLK-1 and another matches to the truncated copy trRLK-2 in A17. This indicated that trRLK-2 is truncated in A17 but not in F83005.5 (Figure 3D). We also found that the RLK-2 is missing in the A20 genome. Further genome sequencing between the two tandem copies revealed an insertion/deletion polymorphism between the three genomes (Figure 3D). The exact size of the insertion in F83005.5 is currently unknown. We are in the process of testing the candidate genes including RLK-2, but our initial experiments showed that transgenic alfalfa and *M. truncatula* roots containing RLK-2 failed to restrict nodulation by Rm41. Thus, it is likely that *NS1* resides in the insertion of F83005.5 and the identity is unknown. The insertion size could be very large because our long-range PCR experiments were unsuccessful to fill the gap. Further de-novo resembling of sequences from other genotypes is required to resolve this issue.

## Conclusions

Establishing a successful interaction between legumes and rhizobia requires signal recognition between the two symbiotic partners. Thus, the evolution of symbiosis specificity involves both rhizobial and host genes. From the bacterial side, specificity determinants include Nod factors, surface polysaccharides, and secreted proteins. However, we know relatively less from the host side. Perception of the Nod-factor signal is mediated by direct binding to the host Nod factor receptors (NFRs), which are plasma membrane-localized receptor kinases containing LysM motifs in their extracellular domains [49]. The role of NFRs in regulating host specificity was demonstrated by transferring the *L. japonicus* versions of *NFRs* to *M. truncatula*, which enabled the transgenic *M. truncatula* roots to nodulate with the *L. japonicus* symbiont *Mesorhizobium loti*[24]. However, natural variation in NFRs that causes changed specificity has rarely been documented. In contrast, numerous dominant genes have been identified in soybeans and other legumes that restrict nodulation with specific rhizobial strains [20,30]. We recently cloned the two soybean genes *Rj2* and *Rfg1* that restrict nodulation with specific strains of *Bradyrhizobium japonicum* and *Sinorhizobium fredii*, respectively [20]. We demonstrated that *Rj2* and *Rfg1* are allelic genes encoding a member of the Toll-interleukin receptor/nucleotide-binding site/leucine-rich repeat (TIR-NBS-LRR) class of plant resistance (R) proteins. Our discovery is consistent with recent reports that documented a large number of secreted effectors delivered into the host cell by rhizobial T3SS and suggests that establishment of a root nodule symbiosis requires the evasion of plant immune responses triggered by rhizobial effectors.

In this study, we have identified a naturally occurring dominant gene, *Mt-NS1*, in *M. truncatula* that prevents nodulation with *S. meliloti* Rm41 and made significant progress toward cloning the gene. The lack of T3SS-encoding genes in the Rm41 genome suggests that *Mt-NS1* unlikely encode an *R* gene. Consistent with prediction, we did not identify typical *R* gene homologs around the *Mt-NS1* locus. We hypothesize that *Mt-NS1* likely encodes a pattern recognition receptor that mediates specific recognition of yet unknown MAMPs of the rhizobial bacteria, resulting in host defense responses. Thus, cloning and characterization of *Mt-NS1* will provide novel insights into the genetic control of nodulation specificity in the legume-rhizobial symbiosis.

## Methods

### Bacterial strains and growth media

The rhizobial strains used in this study included wild-type *S. meliloti* strains NGR34 [50], NGR247 [50], and Rm41 [37] plus several mutant strains of Rm41: AK631 (*exoB631*, ESP-), PP674 (*exoB631*/*rpkA*::Tn5, EPS-KPS-), and PP4409 (*rpk1*::Tn5, KPS-) [51,52]. The Rm41 strain used for fluorescence microscopy contained pHC60 [42], a stably maintained plasmid that constitutively expresses GFP. Strains were grown in TY agar medium at 28°C [53]. Antibiotics were used at 50 μg/ml for spectinomycin and 200 μg/ml for neomycin.

### Plant growth and nodulation assay

*M. truncatula* genotypes used in this study were listed in Table 1. Two F2 populations were developed for genetic mapping of *Mt-NS1*; one was derived from the cross between A20 and F83005.5 and another from the cross of DZA045.5 x F83005.5. Seedlings of parents and the segregating populations were grown in a 50–50 mixture of vermiculite and Turface in a growth chamber programmed for 16 h light at 22°C and 8 h dark at 20°C. The plants were grown under nitrogen-free conditions. For nodulation assay, roots of one-week-old seedlings were inoculated with rhizobial bacteria; each plant was flood-inoculated with 1 ml of a cell suspension with an optical density at 600 nm of 0.1. Nodulation phenotypes were recorded three weeks post inoculation.

### Genetic mapping

For genetic mapping, we first used SSR (simple sequence repeat) markers with known genetic position to localize the approximate position of *Mt-NS1*. Additional markers were then developed based on genomic sequence surrounding the *Mt-NS1* locus. Markers were based on SNPs (single nucleotide polymorphisms) identified between the two parents. For this purpose, primers were designed for PCR amplification of genomic DNA from the two parents of the F2 mapping population, followed by sequencing the PCR products to identify sequence polymorphisms. Where possible, SNPs were converted to CAPS (cleaved amplified polymorphic sequences) markers for genotyping; otherwise, they were genotyped by direct sequencing.

### Fluorescence microscopy

For fluorescence microscopy, roots inoculated with GFP-expressing Rm41 were examined for infection thread formation 5–7 days post inoculation using a FV1000 point-scanning/point-detection laser scanning confocal microscope (Olympus). Before microscopic analysis, the roots were counterstained with 10 mg/ml propidium iodide for 1 minute. After staining, roots were quickly dipped in distilled water to rinse, and mounted under a coverslip. The fluorescence excitation is 488 nm for GFP, and 535 nm for propidium iodide; GFP emission was captured and collected at 520 ± 15 through GFP filter; propidium iodide emission was detected in another channel using a 520 ± 15 nm bandpass. Image acquisition was performed at a resolution of 512 × 512 pixels and a scan rate of 20 μs pixel-1. The FLUOVIEW 1.5 software (Olympus) was used to control the microscope and export images. To examine the bacterial colonization in cortical cells, the nodules or nodule primordia were sliced longitudinally to 100-300 μm thickness before microscopic analysis.

## Competing interests

The authors declare that they have no competing interests.

## Authors’ contributions

HZ conceived the project. JL, SY, and QZ carried out the experiments. HZ wrote the manuscript. All authors read and approved the final manuscript.
